# Draft Genome Sequence of Klebsiella pneumoniae Strain ICIS-278_PBV, Isolated from the Feces of a Healthy 59-Year-Old Man from Orenburg, Russia

**DOI:** 10.1128/genomeA.00576-18

**Published:** 2018-07-05

**Authors:** S. V. Andryuschenko, I. A. Zdvizhkova, N. B. Perunova, O. V. Bukharin

**Affiliations:** a Institute for Cellular and Intracellular Symbiosis of Ural Branch of Russian Academy of Sciences, Orenburg, Russia

## Abstract

This report describes the draft genome sequence of Klebsiella pneumoniae strain ICIS-278_PBV, isolated from the feces of a healthy 59-year-old man from Orenburg, Russia. The size of the genome was 5,584,615 bp (57.2% G+C content). Annotation revealed 5,302 coding sequences, including 5,254 proteins, 23 rRNA genes, and 81 tRNA genes.

## Abstract (precis)

This report describes the draft genome sequence of Klebsiella pneumoniae strain ICIS-278_PBV, isolated from the feces of a healthy 59-year-old man from Orenburg, Russia. The size of the genome was 5,584,615 bp (57.2% G+C content).

## GENOME ANNOUNCEMENT

Klebsiella spp. are some of the main pathogens of nosocomial infections (1). Klebsiella bacteria remain in the top five pathogens of nosocomial pneumonia and urinary tract infections, causing up to 8% of all hospital-acquired infections (2, 3). In particular, Klebsiella spp. cause up to 17% of urinary tract infections, and as causative agents of Gram-negative bacteremia, Klebsiella spp. are second only to Escherichia coli (4, 5).

Historically, Klebsiella pneumoniae has caused serious infections primarily in immunocompromised individuals, but the recent emergence and spread of hypervirulent strains have broadened the number of people who are susceptible to infections, including those who are healthy and immunosufficient. Furthermore, K. pneumoniae strains have become increasingly resistant to antibiotics, rendering infections caused by these strains very challenging to treat (5).

Here, we present a draft genome sequence of Klebsiella pneumoniae strain ICIS-278_PBV, isolated from the feces of a healthy 59-year-old man from Orenburg, Russia.

Preparation of DNA libraries and sequencing were conducted in the Center of Shared Equipment “Persistence of microorganisms” of the Institute for Cellular and Intracellular Symbiosis of the Ural Branch of the Russian Academy of Sciences (RAS; Orenburg, Russia). Genomic DNA was extracted from an overnight culture of K. pneumoniae ICIS-278_PBV and used to prepare a DNA library with the Nextera DNA library preparation kit (Illumina, Inc.). The library was sequenced in a 2 × 300-nucleotide run using the MiSeq reagent kit version 3 and MiSeq desktop sequencer (Illumina, Inc.). The reads were quality trimmed using the sliding window mode of the Trimmomatic program (6). *De novo* genome assembly was performed using the SPAdes genome assembler (St. Petersburg genome assembler, version 3.9.0) (7). The assembly yielded 211 contigs covering a total of 5,584,615 bp with an *N*_50_ value of 91,794 bp, a G+C content of 57.2%, and an average coverage of 34.5×. Six contigs were less than 200 bp and were removed from the analysis. The genome sequence was annotated using the National Center for Biotechnology Information (NCBI) Prokaryotic Genome Annotation Pipeline (PGAP) (https://www.ncbi.nlm.nih.gov/genome/annotation_prok), which revealed 5,570 coding sequences, including 5,302 proteins, 153 pseudogenes, 23 rRNA genes (5S, 16S, and 23S), 81 tRNA genes, and 11 noncoding RNA (ncRNA) genes.

Analysis of the strain K. pneumoniae ICIS-278_PBV genome revealed genes involved in pathogenicity to human hosts, such as those for periplasmic lysozyme inhibitor (8) and aerobactin and colibactin production. The biochemical method ENTEROtest 24 (Pliva-Lachema s.r.o., Czech Republic) revealed that the K. pneumoniae strain ferments glucose, dulcitol, raffinose, melibiose rhamnose, sorbitol, esculin, acetoin, mannitol, trehalose, sucrose, cellobiose, adonitol, inositol, malonate, lysine, and urea.

### Accession number(s).

This whole-genome shotgun project has been deposited at DDBJ/ENA/GenBank under the accession number NCVU00000000. The version described in this paper is version NCVU01000000.
